# Should artificial intelligence have lower acceptable error rates than humans?

**DOI:** 10.1259/bjro.20220053

**Published:** 2023-04-13

**Authors:** Anders Lenskjold, Janus Uhd Nybing, Charlotte Trampedach, Astrid Galsgaard, Mathias Willadsen Brejnebøl, Henriette Raaschou, Martin Høyer Rose, Mikael Boesen

**Affiliations:** 1 Department of Radiology, Bispebjerg-Frederiksberg Hospital, University of Copenhagen, Copenhagen, Denmark; 2 Radiological Artificial Intelligence Testcenter, Copenhagen, Denmark; 3 Department of Psychology, University of Copenhagen, Copenhagen, Denmark; 4 Department of Radiology, Herlev-Gentofte Hospital, University of Copenhagen, Copenhagen, Denmark; 5 Charlie Tango, Copenhagen, Denmark

## Abstract

The first patient was misclassified in the diagnostic conclusion according to a local clinical expert opinion in a new clinical implementation of a knee osteoarthritis artificial intelligence (AI) algorithm at Bispebjerg-Frederiksberg University Hospital, Copenhagen, Denmark. In preparation for the evaluation of the AI algorithm, the implementation team collaborated with internal and external partners to plan workflows, and the algorithm was externally validated. After the misclassification, the team was left wondering: what is an acceptable error rate for a low-risk AI diagnostic algorithm? A survey among employees at the Department of Radiology showed significantly lower acceptable error rates for AI (6.8 %) than humans (11.3 %). A general mistrust of AI could cause the discrepancy in acceptable errors. AI may have the disadvantage of limited social capital and likeability compared to human co-workers, and therefore, less potential for forgiveness. Future AI development and implementation require further investigation of the fear of AI’s unknown errors to enhance the trustworthiness of perceiving AI as a co-worker. Benchmark tools, transparency, and explainability are also needed to evaluate AI algorithms in clinical implementations to ensure acceptable performance.

Will it be easier to forgive your human co-worker than your AI co-worker when they make mistakes? We must face the challenge of comparing computer models to previous human labour as automation and artificial intelligence (AI) may play a substantial role in future radiological workflows. The size of the challenge is currently not yet defined, but it is assumed to grow with the considerable increased number of approved AI algorithms.^1^ Furthermore, automated AI workflows in Radiology without humans in the loop will amplify the challenge. We are moving into an unknown territory where AI methodology will replace human reasoning and set a new standard for good medical practice in all aspects of Radiology.

As an AI research group at the Department of Radiology at Bispebjerg-Frederiksberg University Hospital, in Copenhagen, Denmark, we stumbled upon a crucial question during the first day of clinical implementation: how often may the implemented AI algorithm make mistakes? In our case, a low-risk AI algorithm misclassified the first patient in the knee osteoarthritis diagnostic conclusion for no obvious reasons, and it surprised the implementation group how sceptical they became after the misinterpretation by the AI algorithm. The AI algorithm was implemented in an intended automated triage workflow without immediate clinical supervision. A correct knee osteoarthritis diagnosis is not a life-threatening situation, but an accurate diagnosis still directs the patient to the proper treatment from the beginning. Furthermore, the algorithm avoids potentially inappropriate MRI scans^2^ and arthroscopies^3^ in patients with radiographic knee osteoarthritis.

## Does AI perform as required?

A recent paper^4^ raised two questions when evaluating an AI algorithm: ‘can the machine perform?’, which is often well documented, and ‘will the machine perform as required?’, which is less investigated. When medical devices are evaluated on retrospective data, healthcare providers look at performance metrics, such as sensitivity, specificity, accuracy, and ROC curves. However, these metrics cannot be used if the AI algorithm is not developed or evaluated for the specific clinical purpose. The implementation team had spent almost a year planning the workflows with automatised AI decision-making steps with collaborating external partners from primary care and orthopaedic surgery and internal partners within the radiology department. Furthermore, the AI algorithm had been locally validated on external data with satisfying results compared to local musculoskeletal (MSK) radiology experts.^5^ After noticing the misclassification, our first aim was to find a threshold for an acceptable error rate of the AI algorithm in a clinical setting. The intended use of the algorithm was as an assisted medical device, but in this case, the department used it as a standalone device for triaging patients without immediate clinical supervision. All radiographs and AI reports were later read by a MSK radiologist or trained MSK reporting radiographer.

## Our survey

**Figure 1.**
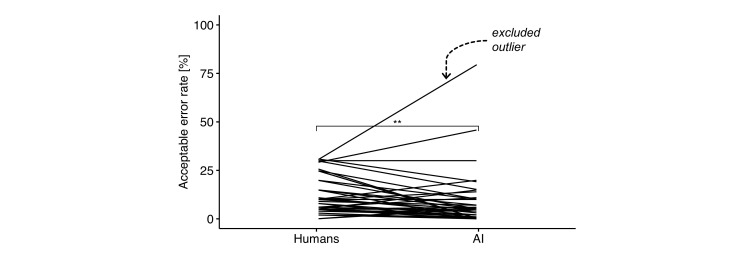
The acceptable error rate in per cent for humans and AI from the survey (*n* = 45). Forty-four participants were included and represented by lines, and there was a significantly lower acceptable error rate for AI than humans (*p* = 0.002, **). The excluded outlier is marked with an arrow.

In order to find an acceptable error rate for standalone devices, the implementation group asked the attendees at a morning conference in February 2022 at the Department of Radiology (radiologists, radiographers, secretaries, and administrative staff) at Bispebjerg-Frederiksberg Hospital the following questions using the informal survey app, Mentimeter, Sweden, for anonymous polls: “How often may reporting radiographers and radiologists make mistakes when reporting on knee osteoarthritis?”. And then, the attendees were asked: “How often may AI models make mistakes when reporting on knee osteoarthritis?”. Participation was voluntary, and the staff was already familiar with AI and the implementation of automated AI workflows. The AI literacy was overall high after continuous AI education to all employees, from secretaries to head of department. Forty-five staff members completed both questions. One participant was excluded as an outlier with a z-score of 4.5, calculated for the difference in rates between the two questions, see Figure 1. The excluded participant had one of the highest acceptable error rates for both humans (30 %) and AI (80 %). The acceptable error rate for AI (n: 44, mean: 6.8 %, SD: 8.8 %) was found to be significantly lower than for humans (n: 44, mean: 11.3 %, SD: 9.1 %) with paired t-test (*p* = 0.002, effect size *r* = 0.5); analysed with R v4.2.0.

## How often do humans make mistakes?

Previous studies have found a 3–6% human error rate on general radiographic examinations^6^ and a 10–14% on knee osteoarthritis binary scoring compared to experts in a controlled research environment.^5^ The same AI algorithm that failed in our implementation had a 13% error rate in an external validation.^5^ These rates are higher than our co-workers accepted for AI in our survey, but on par with the accepted human reader error rate. The discrepancy could be explained by a general lack of confidence in performance of AI.

## Forgiving machines as we forgive humans?

Our study highlights a potential discrepancy between acceptable error rates for AI algorithms and for humans. Are humans afraid of being replaced by machines and expressing their feelings by having a lower threshold of acceptable error for machines? Or is it due to fear and scepticism of AI among healthcare workers?^7,8^ The unpredictability, fear of unknown errors and “black-box” decision-making process of AI algorithms can lead to mistrust. In our case, the AI failure would probably not have created the same tumult if it was a human error. Presumably, humans forgive each other, especially at the beginning of a new workflow or task. Our human co-workers have invested some social capital and likeability, which leaves AI as “the asocial guy in the room.” A previous study has shown that humanoid robots can enhance trustworthiness in a non-healthcare workspace setting.^9^ Trust in AI is threatened by inexplainable outcomes that are counterintuitive for humans. Structural restrictions in the algorithms, together with intermediate calculations, decision trees, and post hoc approximations, can make AI more explainable and show the clinician how the AI came to its conclusions. Humanizing AI in combination with transparency and explainability could be the answer to gaining trustworthy AI. Although not everyone considers AI valuable support, some radiologists fear losing their skills and jobs to AI.^10^ Furthermore, humans have a learning curve, but non-adaptable AI algorithms have no learning curve. FDA-approved AI medical devices are usually “locked” and non-adaptable,^11^ and therefore, they cannot learn from their mistakes. These AI algorithms give the same result for the same radiograph on day one as on day 100. If health authorities in the future will allow adaptable AI, including human-in-the-loop, the discussion of error rates might change and create more sympathy and forgiveness for the AI algorithms. Today, AI algorithms analyse data in the exact same way each time with standardised and structured readings. There is no reader fatigue, and AI can be applied at any hour of the day. Therefore, replacing humans with AI seems convenient and a good choice especially for simple, low-risk, and high-volume tasks. There are other reasons to add AI to a radiological workflow than increasing the accuracy, for example, lowering the reading times or workload burden for the radiologists. The estimated number of radiologists in the future is insufficient,^12^ forcing the radiology departments to decide if compromising potential higher error rates by AI would be acceptable to lower the workload burden. In our implementation, AI takes care of high-volume and low-risk knee osteoarthritis diagnostics and allows the radiologist to interpret more complicated tasks such as cross-sectional examinations. This leaves us questioning ourselves: are machines worth it if their accuracy is slightly worse than humans, but can reduce the increasing workload? Is lower performance also acceptable if AI can lower the cost compared to human readings? And finally, do we tolerate if AI and humans are not making similar mistakes but at the same rate? There is a need for further quantitative and qualitative investigation to continue the discussion of appropriate AI implementation in a clinical environment. This survey was the first step in addressing this critical issue, but it had some limitations. Participants required a smartphone, the study did not include a cross-over design, participant profile information was not registered, and the sample size was not large enough to show a normal distribution without excluding an outlier. The generalisability is limited to other technology-proficient departments with high AI literacy. Slow-adapting departments may experience lower trustworthiness in AI.

## Can we create AI tools that are better than humans if we measure their performance against humans?

Does an error rate always reflect mistakes? For example, the diagnostic criteria for knee osteoarthritis on radiographs are still debated if they represent the clinical condition appropriately.^13^ And even experts in the field can end up with different radiographic diagnostic conclusions.^5^ Can we deduce an error rate from questionable diagnostic criteria? Furthermore, some supervised AI algorithms are trained in uncontrolled environments by letting the algorithms identify patterns in large image datasets. These patterns may not be visible or yet discovered by humans, and therefore, the diagnostic precision of AI algorithms might be higher than the human eye. The number of approved AI algorithms is growing each year,^1^ and radiologists and radiographers must face new types of both true outcomes and errors made by AI. Benchmark evaluation tools where we measure and review inexplainable or surprising outcomes as well as high AI literacy among healthcare providers are needed to ensure patient and provider safety, maintain trust in AI and keep AI developers accountable for their medical devices. Some inexplainable errors may originate from an overgeneralised approach in the development practice of AI medical devices, which could be considered unsound. Transparent and explainable AI with fallback processes is recommended, along with the possibility, to some degree, for custom adaptation of the AI medical device, allowing the retraining of the algorithm for specific clinical settings. Should AI, in general, have lower acceptable error rates than humans? Not necessarily, if they can maintain reasonable standards and support less staffed departments in the future.

## References

[1] Muehlematter UJ, Daniore P, Vokinger KN . Approval of artificial intelligence and machine learning-based medical devices in the USA and Europe (2015-20): a comparative analysis. Lancet Digit Health 2021; 3: e195–203. doi: 10.1016/S2589-7500(20)30292-2 33478929

[2] Menashe L, Hirko K, Losina E, Kloppenburg M, Zhang W, Li L, et al . The diagnostic performance of MRI in osteoarthritis: a systematic review and meta-analysis. Osteoarthritis Cartilage 2012; 20: 13–21. doi: 10.1016/j.joca.2011.10.003 22044841 PMC3934362

[3] Katz JN, Arant KR, Loeser RF . Diagnosis and Treatment of Hip and Knee Osteoarthritis: A Review. JAMA 2021; 325: 568–78. doi: 10.1001/jama.2020.22171 33560326 PMC8225295

[4] Lewis PR, Marsh S . What is it like to trust a rock? A functionalist perspective on trust and trustworthiness in artificial intelligence. Cognitive Systems Research 2022; 72: 33–49. doi: 10.1016/j.cogsys.2021.11.001

[5] Brejnebøl MW, Hansen P, Nybing JU, Bachmann R, Ratjen U, Hansen IV, et al . External validation of an artificial intelligence tool for radiographic knee osteoarthritis severity classification. Eur J Radiol 2022; 150: 110249. doi: 10.1016/j.ejrad.2022.110249 35338955

[6] Robinson PJ, Wilson D, Coral A, Murphy A, Verow P . Variation between experienced observers in the interpretation of accident and emergency radiographs. Br J Radiol 1999; 72: 323–30. doi: 10.1259/bjr.72.856.10474490 10474490

[7] Gaube S, Suresh H, Raue M, Merritt A, Berkowitz SJ, Lermer E, et al . Do as AI say: susceptibility in deployment of clinical decision-aids. NPJ Digit Med 2021; 4(1): 31. doi: 10.1038/s41746-021-00385-9 33608629 PMC7896064

[8] Strohm L, Hehakaya C, Ranschaert ER, Boon WPC, Moors EHM . Implementation of artificial intelligence (AI) applications in radiology: hindering and facilitating factors. Eur Radiol 2020; 30: 5525–32. doi: 10.1007/s00330-020-06946-y 32458173 PMC7476917

[9] Weidemann A, Rußwinkel N . The role of frustration in human-robot interaction-what is needed for a successful collaboration? Front Psychol 2021; 12: : 640186. doi: 10.3389/fpsyg.2021.640186 33868112 PMC8044935

[10] Huisman M, Ranschaert E, Parker W, Mastrodicasa D, Koci M, Pinto de Santos D, et al . An international survey on AI in radiology in 1,041 radiologists and radiology residents Part 1: fear of replacement, knowledge, and attitude. Eur Radiol 2021; 31: 7058–66. doi: 10.1007/s00330-021-07781-5 33744991 PMC8379099

[11] FDA Proposed Regulatory Framework for Modifications to Artificial Intelligence/Machine Learning (AI/ML)-Based Software as a Medical Device (SaMD) - Discussion Paper and Request for Feedback. (2019).

[12] The Royal College of Radiologists. RCR Clinical radiology census report 2021. (2022).

[13] Kohn MD, Sassoon AA, Fernando ND . Classifications in brief: kellgren-lawrence classification of osteoarthritis. Clin Orthop Relat Res 2016; 474: 1886–93. doi: 10.1007/s11999-016-4732-4 26872913 PMC4925407

